# Evaluation of a Drama-Based Experiential Learning Group Programme for Multidisciplinary Staff and People With Lived Experience in Psychiatry

**DOI:** 10.1192/bjo.2022.79

**Published:** 2022-06-20

**Authors:** Rupal Dave, Thomas Walker, Hugh Grant-Peterkin, Robert Fisher, Frank Rohricht

**Affiliations:** 1 East London NHS Foundation Trust, London, United Kingdom; 2 North East London NHS Foundation Trust, London, United Kingdom

## Abstract

**Aims:**

Experiential learning, such as simulation-based training, is widely used in health education. Dramatic self-expression adds another layer through enacted perspective taking, and embodied self-exploration of interaction with others, to foster situated learning. We describe the evaluation of an innovative drama-based experiential learning project involving collaboration between multidisciplinary mental healthcare staff and people with lived experience of mental illness. The programme was facilitated at East London NHS Foundation Trust by a theatre company experienced in delivering workshops with service users. A weekly group programme took place online over 8 weeks during the COVID-19 pandemic and included activities of improvisation, embodied enactments and debriefing. The programme led to co-production of a drama piece that was filmed and distributed online. It was hypothesised that the experiential learning might result in individual benefits for all participants, such as improved well-being and increased mutual understanding of each other's experience of mental health care. The project aimed to improve relationships between healthcare disciplines, and between staff and service users. Additionally, aims were to empower service users, and support staff to practice core interpersonal skills. Objectives of the evaluation were to study the impact of the experiential learning, understand participants’ experience, and explore challenges and benefits.

**Methods:**

A mixed methods approach was taken to evaluate the programme. Following completion of the project, participants were invited to complete a questionnaire utilising a Likert scale rating of overall satisfaction with the project, perceived benefit and impact on specific domains such as working with others. One-to-one semi-structured interviews were conducted according to a topic-guide, and qualitative data were analysed using open & axial coding for thematic analysis.

**Results:**

11 participants, including Psychiatrists, Occupational Therapists and current service users, completed the experiential learning and filming. Questionnaire data suggested participants were highly satisfied with the learning and felt it would be valuable to others. Themes include the positive experience of creativity, dismantling of hierarchy, improved empathy, confidence and connection. Potential challenges were digital inequality and lack of dedicated time for professional development.

**Conclusion:**

A drama-based experiential learning group programme for healthcare staff and service users is a highly beneficial learning experience. Participants describe changes on a personal level as well as improved understanding of others’ perspectives. This form of experiential learning features collaborative working that aligns with principles of co-production and supports the development of interpersonal skills; the findings suggest that drama-based experiential learning is a useful method in health education to complement knowledge acquisition.

